# Late gadolinium enhancement by cardiovascular magnetic resonance is complementary to left ventricle ejection fraction in predicting prognosis of patients with stable coronary artery disease

**DOI:** 10.1186/1532-429X-14-29

**Published:** 2012-05-19

**Authors:** Oronzo Catalano, Guido Moro, Mariarosa Perotti, Mauro Frascaroli, Monica Ceresa, Serena Antonaci, Paola Baiardi, Carlo Napolitano, Maurizia Baldi, Silvia G Priori

**Affiliations:** 1 Divisione di Cardiologia, IRCCS Fondazione Salvatore Maugeri, via Maugeri 6, Pavia, Italy; 2 Servizio di Diagnostica per Immagini, IRCCS Fondazione Salvatore Maugeri, via Maugeri 6, Pavia, Italy; 3 Divisione di Cardiologia, Presidio Ospedaliero Sacro Cuore, Gallipoli, Italy; 4 Direzione Scientifica Centrale, IRCCS Fondazione Salvatore Maugeri, via Maugeri 6, Pavia, Italy; 5 Unità di Cardiologia Molecolare, IRCCS Fondazione Salvatore Maugeri, via Maugeri 6, Pavia, Italy; 6 The Leon Charney Division of Cardiology, New York University School of Medicine, New York, USA; 7 Dipartimento di Cardiologia, Università of Pavia, Pavia, Italy

## Abstract

**Background:**

Late gadolinium enhancement (LGE) cardiovascular magnetic resonance (CMR) predicts adverse prognosis in patients with stable coronary artery disease (CAD). However, the interaction with conventional risk factors remains uncertain. Our aim was to assess whether the extent of LGE is an independent predictor of adverse cardiac outcome beyond conventional risk factors, including left ventricle ejection fraction (LVEF).

**Methods:**

We enrolled 376 patients (88% males, 64 ± 11 years) with stable CAD, who underwent LGE assessment and a detailed conventional evaluation (clinical and pharmacological history, risk factors, ECG, Echocardiography). During a follow-up of 38 ± 21 months, 56 events occurred (32 deaths, 24 hospitalizations for heart failure).

**Results:**

LGE and LVEF showed the strongest univariate associations with end-points (HR: 13.61 [95%C.I.: 7.32-25.31] for LGE ≥ 45% of LV mass; and 12.34 [6.80-22.38] for LVEF ≤ 30%; *p* < 0.0001). Multivariate analysis identified baseline LVEF, loop diuretic therapy, moderate-severe mitral regurgitation and pulmonary hypertension as significant predictors among conventional risk factors. According to a step-wise approach, LGE showed strong association with prognosis as well (5.25 [2.64-10.43]; *p* < 0.0001). LGE significantly improved the model predictability (chi-square 239 vs 221, F-test *p* < 0.0001) with an additive effect on the prognostic power of LVEF, which however retained its prognostic power (4.89 [2.50-09.56]; *p* < 0.0001). Patients with LGE ≥ 45% and/or LVEF ≤ 30% had much worse prognosis compared to patients without risk factors (annual event rates of 43% vs 3%; *p* < 0.0001). Interestingly LGE was a significant predictor when all cause mortality was analyzed as the only endpoint.

**Conclusions:**

This study demonstrates that LGE assessed by CMR is a robust independent non-invasive marker of prognosis in stable CAD patients. LGE can integrate the available metrics to substantially improve risk stratification.

## Background

Late gadolinium enhancement (LGE), assessed with cardiovascular magnetic resonance (CMR), has high sensitivity and specificity to detect and quantify fibrotic tissue due to myocardial infarction (MI) [1-3].

Previous studies suggested that LGE predicts adverse prognosis in patients with stable coronary artery disease (CAD) [4-9]. Since it also predicts unfavourable left ventricle (LV) remodelling after acute MI [10], LGE might be considered a metric of LV pump dysfunction alternative to ejection fraction (EF) or end-systolic volume (ESV). Pathophysiologic correlation between LGE and LVEF and equivalence of informative content seem to be supported by studies showing that LGE inclusion in multivariate models often leads to substantial blunting of the well known prognostic power of LVEF [5,6]. Interestingly, however, this evidence has not been confirmed by other studies in which both scar extent and LVEF seem to have an independent prognostic value [9,11]. Moreover, LGE retains a prognostic significance in the subset of patients with reduced LVEF [7]. Finally, conflicting results have been found in patients with stable CAD and unrecognized MI, with LGE prognostic power being incremental or alternative to LVEF at multivariate analysis [4,8]. Thus, the prognostic significance of LGE seems to be complex and not yet completely elucidated.

The aim of our study was to assess whether, in a large well-characterized population of patients with known or suspected stable CAD the extent of LGE is an independent predictor of adverse outcome in the long-term beyond conventional risk factors, in particular LVEF.

## Methods

### Study population and design

We performed a single centre observational prospective study. Inclusion criteria: consecutive patients clinically referred for CMR from January 2002 to December 2006, either with definite diagnosis or with a history suggesting stable CAD. Exclusion criteria: recent acute coronary syndrome (within 6 weeks), previous hospitalization for heart failure (NYHA class IV or need of infusive therapy) and signs of myocarditis, infiltrative or hypertrophic cardiomyopathy and pericardial disease. Patients underwent detailed clinical and instrumental risk stratification and were prospectively followed up. The study was approved by the Fondazione Maugeri ethical committee and informed consent was obtained from patients.

### Conventional risk assessment

Before CMR execution patients’ clinical history was collected, including anthropometric data, atherosclerotic risk factors profile, any documented CAD history, coronary angiography, NYHA class and pharmacological records. An ECG was also recorded the same day of CMR. ECGs were automatically analysed about heart rate, PR interval, QTc interval and QRS duration (E-Scribe System, Mortara Rangoni Europe), and interpreted by a single blinded reader (O.C.) with regard to rhythm, signs of LV hypertrophy, left and right bundle branch block, ST depression, negative T waves and Q waves presence. Patients underwent an echocardiographic examination within few days from CMR with up-to-date equipments (Sonos 5500, Hewlett Packard; Sequoia 512, Acuson; Vivid 7, General Electric). We evaluated dimensions, mass, segmental/global contractility and diastolic function of LV, mitral regurgitation, RV dimension and function, and pulmonary artery pressure (evaluation criteria for ECG and Echo are provided in additional file 1).

### CMR and LGE assessment

CMR was performed with a 1.0-T scanner with a 20mTgradient (Magneton Harmony, Siemens, Erlangen, Germany) and a phased-array cardiac coil. LGE was assessed by inversion-recovery turboFLASH sequences (TE 2.6 msec, FA 8°, inversion time 260–360 msec, matrix 96 × 256; FOV 400 mm), 7–8 min after 0.15–0.20 mmol/kg intravenous injection of gadolinium (Magnevist; Schering, Berlin, Germany; Multihance, Bracco, Milan, Italy). Multiple 8 mm thick short-axis slices (usually 8–10) with appropriate interslice space (usually 2 mm) were used for a full coverage of LV. Transmural extent of LGE was scored by consensus of two experienced readers (O.C., G.M.), using a five point scale: 0 = no LGE, 1 = 1–25%, 2 = 26–50%, 3 = 51–75% and 4 = 76–100% of wall thickness [12]. Standard 17-segments segmentation of LV was used and total LGE burden in percent of total LV mass was calculated ([total score*100]/[17*4]). Maximal transmural extent and spatial extent, that is maximal LGE score and the number of affected segments, were considered too.

### Follow-up

Follow up visits were performed at our Centre every 1–24 months, depending on the clinical severity. Trans-telephonic follow-up was collected only for those patients whose last visit date was antecedent 6 month database closure (March 2009).

Primary outcome measure was a composite clinical end-point of all-cause mortality and new onset heart failure (HF) requiring hospitalization (NYHA class IV or need of infusive therapy). If the patients were admitted to a hospital other than our institution we retrieved the hospital record to confirm diagnosis, clinical parameters and outcome.

Myocardial revascularization procedures occurring after CMR were registered as well, to evaluate any modulating effect of myocardial revascularization on prognostic value of LGE.

### Statistics

Categorical variables were expressed as counts and percentage, continuous variables as mean ± standard deviation. Two sided *P* < 0.05 was the significance level for hypothesis testing and SPSS Statistics 18.0 was the statistical package we used.

Differences at baseline between patients with and without events were tested with Pearson Chi-Square or Fisher's exact test in case of categorical variables and Student's *t*-test or Mann–Whitney *U*-test in case of continuous variables.

Univariate hazard ratios were calculated by Cox analysis after converting continuous variables into dichotomous variables; cut-offs were taken from the literature. Specifically dichotomization of LVEF was made according to the more conservative SCDHeft cut-off (30%). If established cut-offs were lacking, we used the 75th and the 95th percentiles of the entire study population. Proportional hazard assumption was graphically tested using plots of the log estimated cumulative baseline hazard against time.

Conventional variables correlated with prognosis (*p* < 0.1) at a first multivariate analysis (step-wise forward selection, forceful introduction of LVEF), were used to build the final model in which LGE was introduced at the last step to test the hypothesis of its independent prognostic value on top of a conventional risk stratification approach. F-test for extra sum of square principle was applied to assess goodness of fit of the final model with respect to the conventional nested model. Annual event rate and death rate for patients at risk were calculated.

## Results

Four-hundred-ten patients were referred to our unit for CMR assessment during the period of interest. Twenty-seven (7%) were excluded according to the exclusion criteria and seven (2%) patients were lost at follow-up. Thus, 376 patients entered the study, with a definite diagnosis of CAD at the enrolment in 332 cases (88%) and suspicion history in 44 (12%). Patients were followed-up for 38 ± 21 months, during which there were 56 events (32 deaths, 24 new onset HF cases).

Main baseline characteristics are reported in Table 1. Overall the study cohort was characterized by high prevalence of male sex (88%). History of previous MI was detected in two thirds of cases. Pharmacological treatment was characterized by high rates of beta-blockers, ACE-inhibitors, ASA and statins administration (70-85%) with no significant differences among patients with and without events.

**Table 1:** Baseline characteristics and differences between patients with and without events in the follow-up

	**All patients**	**Event free (n = 320)**	**With events (n = 56)**	P Value*
ANTHROPOMETRY				
Age (y)	64 ± 11	63 ± 11	68 ± 10	0.003
Sex (m)	292 (78%)	248 (76%)	44 (79%)	0.859
Body mass index	26 ± 4	26 ± 4	26 ± 4	0.300
CAD RISK FACTORS				
Familiar history of CAD	170 (45%)	143 (45%)	27 (48%)	0.625
Smoking habit	220 (59%)	179 (56%)	41 (73%)	0.015
Diabetes	77 (21%)	66 (21%)	11 (20%)	0.867
Hypertension	218 (58%)	185 (58%)	33 (59%)	0.876
Hypercholesterolemia	214 (57%)	181 (57%)	33 (59%)	0.742
# risk factors	2.4 ± 1.1	2.4 ± 1.1	2.6 ± 1.1	0.159
CLINIC HISTORY				
Previous CAD diagnosis	332 (88%)	277 (87%)	55 (98%)	0.012
Previous myocardial infarction	246 (65%)	202 (63%)	44 (79%)	0.025
NYHA classification (III class)	22 (6%)	11 (3%)	11 (20%)	<0.0001
Revascularization in the follow-up	79 (21%)	73 (23%)	6 (11%)	0.040
PHARMACOLOGICAL THERAPY				
β-blockers	289 (77%)	448 (78%)	41 (73%)	0.483
Ca^++−^antagonist	76 (20%)	62 (19%)	14 (25%)	0.334
Nitrates	159 (42%)	136 (43%)	23 (41%)	0.842
Loop diuretics	135 (36%)	95 (30%)	40 (71%)	<0.0001
Aldosterone antagonist	51 (14%)	30 (9%)	21 (38%)	<0.0001
ACE-inhibitors/AT_1_-receptors antagonist	304 (81%)	257 (80%)	47 (84%)	0.526
ASA	319 (85%)	275 (86%)	44 (79%)	0.156
Statins	280 (75%)	240 (75%)	40 (71%)	0.572
Anticoagulant	33 (9%)	18 (6%)	15 (27%)	<0.0001
ECG				
Heart rate (bpm)	65 ± 13	64 ± 12	73 ± 14	<0.0001
Non sinusal rhythm	12 (3%)	7 (2%)	5 (9%)	0.021
QRS duration (msec)	105 ± 21	103 ± 19	112 ± 27	0.022
QTc interval (msec)	425 ± 34	421 ± 32	447 ± 37	<0.0001
LV hypertrophy	58 (15%)	50 (14%)	8 (16%)	0.942
LBB block	59 (16%)	44 (14%)	15 (27%)	0.013
RBB block	16 (12%)	12 (12%)	4 (14%)	0.612
ST segment depression	46 (8%)	38 (7%)	8 (13%)	0.176
Negative T waves	184 (49%)	151 (47%)	33 (59%)	0.105
Q waves	164 (44%)	135 (42%)	29 (52%)	0.181
ECHOCARDIOGRAPHY				
LV EDV (ml/m^2^)	59 ± 22	57 ± 20	74 ± 30	<0.0001
LV ESV (ml/m^2^)	31 ± 20	28 ± 16	49 ± 28	<0.0001
LV EF (%)	51 ± 13	53 ± 12	39 ± 15	<0.0001
LV WMSI	1.4 ± 0.5	1.4 ± 0.4	1.9 ± 0.5	<0.0001
LV mass (g)	188 ± 59	186 ± 57	202 ± 70	<0.0001
LV diastolic function (≥ pseudo-normal)	44 (12%)	25 (8%)	19 (34%)	<0.0001
Mitral regurgitation (≥ moderate)	56 (15%)	36 (11%)	20 (36%)	<0.0001
Pulmonary hypertension	34 (9%)	19 (6%)	15 (27%)	<0.0001
RVIT dilatation	17 (5%)	12 (4%)	5 (9%)	0.085
RV dysfunction	38 (10%)	28 (9%)	10 (18%)	0.037
LATE GADOLINIUM ENHANCEMENT				
Total burden (% of LV mass)	13 ± 15	10 ± 12	28 ± 22	<0.0001
Spatial extent (% of LV surface)	22 ± 22	18 ± 19	42 ± 29	<0.0001
Max transmural extent (% of wall thickness)	55 ± 39	51 ± 39	73 ± 39	<0.0001

LV = left ventricle; LBB = left bundle branch; RBB = right bundle branch; MR = mitral regurgitation; RVIT = right ventricle inflow tract.

* Pearson Chi-Square or Fisher's exact test (where appropriate) for categorical data; Student's *t*-test or Mann–Whitney test (# risk factors, LV WMSI, late enhancement data) for numeric data.

### Predictors of events

Survival Cox univariate analyses showed that outcome was associated with several variables. As known from previous studies, the most powerful predictors of events were age, previous history of CAD, 3-vessel disease at coronary angiography, NYHA class, need for diuretic and anticoagulant therapy, heart rate, non sinus rhythm, QRS complex duration, QTc interval, LV volumes, LVEF, LV wall motion score index, LV diastolic function, mitral regurgitation, pulmonary hypertension and right ventricle function. A revascularization procedure after the study enrolment was a protecting factor against the outcome. All LGE indexes were also strongly associated with prognosis with total burden, that is the amount of LGE in percent of total LV mass, showing the most powerful correlation. The 95th percentiles was the best cut-off and that was considered for further analysis. Univariate hazard ratios with 95% confidence intervals of all considered variables are shown in Table 2.

**Table 2:** Unadjusted hazard ratios for death or new heart failure

	**Unadjusted HR**	**95% Confidence Interval**	**P value**
ANTHROPOMETRIC			
Age (75 y)	1.70	0.92 – 3.17	0.093
Male sex	0.92	0.67 – 1.27	0.614
Body mass index >30	1.04	0.49 – 2.20	0.916
RISK FACTORS			
Familiar history of CAD	1.16	0.68 – 1.96	0.588
Smoking habit	2.22	1.23 – 4.00	0.008
Diabetes	0.88	0.45 – 1.70	0.698
Hypertension	1.00	0.59 – 1.70	0.999
Hypercholesterolemia	1.13	0.66 – 1.92	0.664
# risk factors ≥ 3	1.58	0.93 – 2.69	0.093
CLINIC			
Previous CAD diagnosis	8.76	1.21 – 63.3	0.032
Previous myocardial infarction	2.51	1.32 – 4.76	0.005
NYHA classification ≥ 3	5.81	2.99 – 11.3	<0.0001
Revascularization in the follow-up	0.36	0.15 – 0.85	0.019
THERAPY			
β-blockers	1.00	0.55 – 1.81	0.997
Ca^++−^antagonist	1.15	0.63 – 2.12	0.644
Nitrates	0.92	0.54 – 1.56	0.753
Loop diuretics	5.26	2.94 – 9.40	<0.0001
Aldosterone antagonist	5.69	3.29 – 9.86	<0.0001
ACE-inhibitors/AT_1_-receptors antagonist	1.45	0.71 – 2.96	0.310
ASA	0.69	0.36 – 1.30	0.251
Statins	0.94	0.53 – 1.68	0.837
Anticoagulant	6.38	3.49 – 11.7	<0.0001
ECG			
Heart rate (≥75 bpm)	2.80	1.62 – 4.84	<0.001
Non sinusal rhythm	4.19	1.67 – 10.54	0.002
QRS duration	2.62	1.47 – 4.70	0.001
QTc interval	4.34	2.53 – 7.46	<0.0001
LV hypertrophy	1.00	0.47 – 2.11	0.995
LBB block	1.63	1.07 – 2.48	0.024
RBB block	1.30	0.62 – 2.76	0.489
ST segment depression	1.60	0.73 – 3.54	0.245
Negative T waves	1.76	1.03 – 3.00	0.039
Q waves	1.55	0.92 – 2.62	0.102
ECHOCARDIOGRAPHY			
LV EDV (≥ 105 ml/m^2^)	4.66	2.34 – 9.29	<0.0001
LV ESV (≥ 75 ml/m^2^)	8.95	4.55 – 17.60	<0.0001
LV EF (≤ 30%)	12.34	6.80 – 22.38	<0.0001
LV WMSI (≥ 2.32)	10.94	5.53 – 21.62	<0.0001
LV mass (≥ 310 g)	4.89	2.05 – 11.70	<0.001
LV diastolic function (≥ pseudo-normal)†	7.03	4.00 – 12.38	<0.0001
Mitral regurgitation (≥ moderate)‡	4.67	2.68 – 8.13	<0.0001
Pulmonary hypertension	4.86	2.68 – 8.80	<0.0001
RVIT dilatation	1.45	0.92 – 2.30	0.112
RV dysfunction	5.17	2.48 – 10.80	<0.0001
CMR			
LGE total burden ≥ 45% §	13.61	7.32 – 25.31	<0.0001
LGE total burden ≥ 20% #	6.62	3.86 – 11.38	<0.0001
LGE spatial Extent ≥ 68% §	9.27	4.86 – 17.66	<0.0001
LGE spatial Extent ≥16% #	4.96	2.60 – 9.45	<0.0001
LGE transmurality	4.82	2.81 – 8.31	<0.0001

LV = left ventricle; LVEF = left ventricle ejection fraction; LBB = left bundle branch; RBB = right bundle branch; MR = mitral regurgitation; RVIT = right ventricle inflow tract; LGE = late gadolinium enhancement.

† based on trans-mitral diastolic flow and pulmonary vein flow evaluation.

‡ based on effective regurgitate orifice area.

§ cut-off equal to the 95% percentile of the entire population.

# cut-off equal to the 75% percentile of the entire population.

The step-wise inclusion of variable reaching the predefined univariate p value threshold (*p* < 0.1) into a multivariate Cox model in which LVEF was forcefully included, significantly improved the model predictability (chi-square 163 vs 157, F test: *p* = 0.033) with respect to considering LVEF alone. However, only loop diuretic therapy (HR: 3.20 [95% C.I.: 1.71 – 5.97]; *p* = 0.003), pulmonary hypertension (2.44 [1.27 – 4.70]; *p* = 0.008) and moderate-severe mitral regurgitation (2.02 [95% CI: 1.06 – 3.85]; *p* = 0.028) were independently associated with an adverse prognosis after considering LVEF (5.54 [2.85 – 10.78]; *p* < 0.0001).

### Prognostic role of late gadolinium enhancement

Significant conventional variables as from the multivariate analysis were used to build the final model, that included LGE and showed LGE to be significantly associated with an adverse prognosis in terms of death or new onset HF. LGE introduction significantly improved the model fit (chi-square 238 vs 223, F-test *p* = 0.0001) with respect to conventional variables alone. Moreover, LGE was the strongest prognostic indicator with a 5.3 fold increase of event risk at follow-up . However, LVEF retained a comparable prognostic power with a 4.9 fold increase of event risk (HRs shown in Table 3). Similar results were obtained analysing only patients with MI history at the enrolment (n = 246), with a definite diagnosis of CAD at the end of the study (n = 344) or using CMR derived LVEF; HRs of these analyses are provided in additional file 2.

**Table 3:** Adjusted hazard ratios for death or new heart failure of the final model

	**Adjusted HR**	**95% Confidence Interval**	**P value**
LGE total burden (≥ 45% of LV mass)	5.25	2.64 – 10.43	<0.0001
LVEF (≤ 30%)	4.89	2.50 – 9.56	<0.0001
Pulmonary hypertension (sPAP ≥ 35 mmHg)	2.89	1.56 – 5.36	<0.001
Loop diuretics therapy	2.92	1.54 – 5.55	0.001

LGE = late gadolinium enhancement; LVEF = left ventricle ejection fraction; sPAP = systolic pulmonary artery pressure.

Accordingly, LVEF less than 30% and LGE total burden more than 45% of total LV mass (95th percentile of the study population) sharply stratified the population at risk. The absence of both risk factors identified patients at low risk (n = 340), with event-free survival of 95% at 1 year, 92% at 3 years and 86% at 5 years, and mean annual event rate of 3%; conversely the presence of at least 1 of these risk factors identified patients at very high risk (n = 36) with event-free survival of 60% at 1 year, 31% at 3 years and 21% at 5 years, and a mean annual rate of 43% (Log-Rank test: *p* < 0.0001). Ten out of 36 (28%) patients at high risk would not have been identified without considering LGE, since their LVEF was greater than 30%.

**Figure 1:**
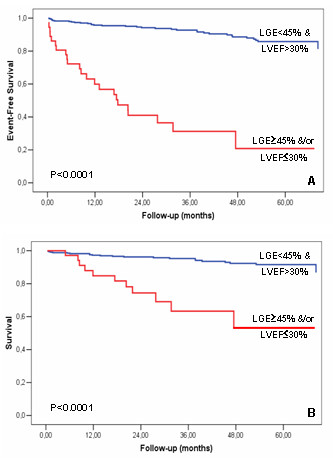
**Prognostic value of late gadolinium enhancement and left ventricle ejection fraction.** Kaplan-Meier survival analysis showing the cumulative incidence of death plus heart failure (panel a) or death alone (panel b).

Finally we repeated the analysis using total mortality as the only endpoint and we found similar results. In this group LGE was associated with significantly increased risk of mortality (HR 3.78 [95% CI: 1.46 – 9.75]; *p* = 0.006). All-cause mortality showed survival of 97% at 1 year, 95% at 3 years and 92% 5 years, and mean annual death rate of 2% in the group without risk factors; compared to survival of 82% at 1 year, 63% at 3 years and 53% at 5 years, and mean annual death rate of 14% in the group with risk factors (*p* < 0.0001). These findings are graphically shown by Kaplan-Meyer curves in the Figure 1.

## Discussion

In the last decade LGE CMR has paved the way to a new era in the assessment of CAD [1]. Indeed, for the first time this technique allowed to pursue a direct detection and quantification of areas of irreversibly injured myocardium. Thus LGE enabled in vivo morphopathologic assessment complementary to that of myocardium functionally impaired but still viable (hibernating myocardium), postulated from previous studies [13]. Accordingly, LGE had the potential to become the technique of choice to study myocardial viability. Recent studies have confirmed this hypothesis by showing that (a) the lesser transmural extent of scar is, the more likely functional recovery of a segment will be after revascularization and (b) the amount of viable plus normal segments is the best predictor of global functional recovery [12,14].

In survival studies the extent of LGE has emerged as the strongest prognostic factor in stable CAD patients with previous MI [5,6]. This correlation further refines the relationship between LVEF and prognosis because LGE directly reflects the amount of irreversibly injured myocardium. Accordingly, these studies seem to support the hypothesis that LVEF is no more significant once LGE is included in multivariate models. On the other hand, the idea of the predictive value of LVEF to be enclosed and overtaken by LGE assessment, is in conflict with a previous scintigraphy study in sudden death survivors and in a recent CMR study in hypertensive patients [9,11]. In these two studies, indeed, LVEF remained an independent predictor of events after the inclusion of scar dimension in a multivariate analysis. Thus the interaction between LGE and LVEF in stable CAD populations seems to be modulated by the selection of specific subsets of patients.

### Conventional risk stratification

Our study was aimed at verifying these findings in a large group of unselected patients with stable CAD. For this reason we intentionally avoid to enrol a highly homogeneous study cohort by defining relatively loose entry criteria. In this setting, we attempted to test the hypothesis whether LGE is an independent predictor of adverse cardiac outcome in the context of a complex cohort undergoing a complete standard evaluation. We believe this study is representative of clinical referral of many outpatient CAD cardiology clinics.

We studied a group of 376 consecutive patients with stable CAD and optimized medical therapy, who were followed-up for an average time of 3 years. The conventional prognostic factor included in the study confirmed their association with adverse prognosis, thus further strengthening the evidence that our study group is representative of a general population with stable CAD. To remove redundant information a multivariate analysis was performed, which confirmed the expected strong prognostic power of LVEF and showed a significant contribution by only few other conventional variables. The latter did not include myocardial revascularization, even if it seemed to have a protective effect as from univariate analysis.

### Added value of late gadolinium enhancement

On top of relevant conventional variables, we found that LGE was significantly and strongly associated with prognosis in a multivariate model (5.3 fold risk increase for LGE >45%). Even more importantly, the present study showed that LGE extent is complementary to LVEF in stratifying the risk of patients with stable CAD. A large scar, replacing more than 45% of total LV mass, well integrates with the generally accepted criterion of LVEF lower than 30% in identifying patients at high risk of death or HF. Accordingly, patients with either LGE ≥ 45% or LVEF ≤ 30%, have completely different prognosis if compared with patients without any of these risk factors, showing annual event rates of 43% versus 3% and mortality rates of 14% versus 2%, respectively. Notably, about 30% of patients at high risk would not have been identified without including LGE into the stratification process.

The result of the present study seems to reinforce the idea of LGE as a predictor of prognosis that adds to LVEF. In comparison to the studies of Roes et al. and Kelle et al., that came to opposite results, our study considered similar end-points but did not have scar presence as an inclusion criterion (approximately 70% of our patients were LGE positive). Accordingly, on average scar dimension was smaller (13% vs 19–20% of total mass) and EF higher (51 vs 43–44%). Thus, it may be hypothesized that the pre-selection of patients with a scar among those with stable CAD may influence the interaction between LGE and LVEF in the prediction of future events.

Understanding the relative relevance of LGE and LVEF is not a purely theoretical issue. Indeed, in patients with stable CAD reduced LVEF is a pre-requisite for important therapeutic decisions, such as cardiac resynchronization therapy (CRT) or automatic implantable cardioverter-defibrillator (AICD). Thus, will LGE be confirmed a stronger predictor than LVEF, the management of patients with stable CAD could be substantially changed

## Conclusions

Our study confirms and refines the evidence of LGE as strong prognostic factor in unselected patients with stable CAD, showing a complementary role with respect to LVEF. Although further studies are warranted to assess the usefulness of LGE as selection criterion for major therapeutic decision such as CRT or AICD, findings of the present study promote the inclusion of LGE into current clinical management of patients with stable CAD, especially of those with reduced LVEF at echocardiography.

## Competing interests

The authors declare that they have no competing interests.

## Authors' contributions

OC conceived and designed the study, evaluated CMR images, performed statistical analyses, interpreted results and wrote the manuscript. GM conceived and designed the study, evaluated CMR images. MP cooperated in the paper drafting. MF cooperated in the data acquisition. MC cooperated in the data acquisition. SA designed the study, cooperated in data analysed and interpreted results. PB critically cooperated in the results interpretation and revised statistics. CN critically revised the manuscript. MB gave the final approval. SGP gave the final approval. All authors read and approved the final manuscript.

## Supplementary Material

Additional file 1 **Table 1.** Evaluation criteria of ECG and Echocardiography. LV = left ventricle; LBB = left bundle branch; RBB = right bundle branch; MR = mitral regurgitation; sPAH = systolic pulmonary artery hypertension; RV = right ventricle; RVIT = right ventricle inflow tract; TAPSE = tricuspid anular plane systolic excursion * based on trans-mitral diastolic flow and pulmonary vein flow evaluation † based on proximal isovelocity surface area radius. Click here for file

Additional file 2 **Table 2.** Adjusted hazard ratios for death or new heart failure of the final model for patients with myocardial infarction history (panel a), with definite diagnosis of coronary artery disease (panel b), and using MR derived left ventricle ejection fraction (panel c). LGE = late gadolinium enhancement; LVEF = left ventricle ejection fraction; sPAP = systolic pulmonary artery pressure. Click here for file
