# Supplementary material for: Late gadolinium enhancement by cardiovascular magnetic resonance is complementary to left ventricle ejection fraction in predicting prognosis of patients with stable coronary artery disease
Source: J Cardiovasc Magn Reson. 2012 May 19;14(1):29. doi: 10.1186/1532-429X-14-29 (PMC3405456; doi:10.1186/1532-429X-14-29)
Supplement: Additional file 1 — Table 1. Evaluation criteria of ECG and Echocardiography. LV = left ventricle; LBB = left bundle branch; RBB = right bundle branch; MR = mitral regurgitation; sPAH = systolic pulmonary artery hypertension; RV = right ventricle; RVIT = right ventricle inflow tract; TAPSE = tricuspid anular plane systolic excursion * based on trans-mitral diastolic flow and pulmonary vein flow evaluation † based on proximal isovelocity surface area radius. [file 1532-429X-14-29-S1.doc]

Table 1 (suppl. data). Evaluation criteria of ECG and Echocardiography

|  |  | **Classification criteria/Cut-offs** | **Reference Category** |
| --- | --- | --- | --- |
|  | ECG |  |  |
|  | Rhythm | sinusal / non sinusal | sinusal |
|  | Heart rate | ≥75 bpm | <75 bpm |
|  | QRS complex enlargement | 120 msec | <120 msec |
|  | QTc interval prolongation | 460 msec | <460 msec |
|  | LV hypertrophy | Cornel or Sokolov criteria | no LV hypertrophy |
|  | LBB block | no or incomplete LBB / complete LBB | no or incomplete LBB |
|  | RBB block | no or incomplete / complete RBB | no or incomplete RBB |
|  | ST segment displacement | ST displacement > 1 mm | no ST displacement |
|  | Inverted T waves | inverted T waves in ≥ 2 contiguous leads | no inverted T waves |
|  | Q waves | > 40 msec in ≥ 2 contiguous leads | no Q waves |
|  | ECOCARDIOGRAPHY |  |  |
|  | LV end-diastolic volume* | ≥105 ml | <105 ml |
|  | LV end-systolic volume* | ≥75 ml | <75 ml |
|  | LV ejection fraction* | ≤30 | >30% |
|  | LV wall motion score index | ≥2.30 | <3.30 |
|  | LV mass | ≥310 g | <310 g |
|  | LV diastolic function† | normal or impaired compliance / pseudonormal or restrictive | normal or impaired compliance |
|  | Mitral regurgitation‡ | no or mild MR / moderate or severe MR | no or mild MR |
|  | Systolic pulmonary artery hypertension | sPAP ≥ 35 mmHg | sPAH<35 mmHg |
|  | RV dilatation | RVIT≥40 mm | RVIT>40 mm |
|  | RV dysfunction | TAPSE ≤18 mm | TAPSE>18 mm |

LV=left ventricle; LBB=left bundle branch; RBB=right bundle branch; MR=mitral regurgitation; sPAH=systolic pulmonary artery hypertension; RV=right ventricle; RVIT=right ventricle inflow tract; TAPSE=tricuspid anular plane systolic excursion

* based on modified Simpson’s rule method

† based on trans-mitral diastolic flow and pulmonary vein flow evaluation

‡ based on based on proximal isovelocity surface area radius
