# Supplementary material for: Late gadolinium enhancement by cardiovascular magnetic resonance is complementary to left ventricle ejection fraction in predicting prognosis of patients with stable coronary artery disease
Source: J Cardiovasc Magn Reson. 2012 May 19;14(1):29. doi: 10.1186/1532-429X-14-29 (PMC3405456; doi:10.1186/1532-429X-14-29)
Supplement: Additional file 2 — Table 2. Adjusted hazard ratios for death or new heart failure of the final model for patients with myocardial infarction history (panel a), with definite diagnosis of coronary artery disease (panel b), and using MR derived left ventricle ejection fraction (panel c). LGE = late gadolinium enhancement; LVEF = left ventricle ejection fraction; sPAP = systolic pulmonary artery pressure. [file 1532-429X-14-29-S2.docx]

Table 2.a (suppl. data). Adjusted hazard ratios for death or new heart failure of the final model for patients with myocardial infarction history

|  | **Adjusted HR** | **95% Confidence Interval** | **P value** |  | |
| --- | --- | --- | --- | --- | --- |
| LGE total burden (≥ 45% of LV mass) | 5.00 | 2.47 – 10.13 | <0.0001 |  | |
| Loop diuretics therapy | 4.54 | 2.06 – 10.01 | <0.001 |  | |
| LVEF (≤ 30%) | 4.23 | 2.10 – 8.48 | <0.0001 |  | |
| LGE= late gadolinium enhancement; LVEF=left ventricle ejection fraction; sPAP= systolic pulmonary artery pressure | | | | |  |

Table 2.b (suppl. data). Adjusted hazard ratios for death or new heart failure of the final model for patients with definite diagnosis of coronary artery disease

|  | **Adjusted HR** | **95% Confidence Interval** | **P value** |  | |
| --- | --- | --- | --- | --- | --- |
| LGE total burden (≥ 45% of LV mass) | 5.20 | 2.62 – 10.30 | <0.0001 |  | |
| LVEF (≤ 30%) | 4.79 | 2.45 – 9.34 | <0.0001 |  | |
| Pulmonary hypertension (sPAP ≥ 35 mmHg) | 3.04 | 1.58 – 5.83 | <0.001 |  | |
| Loop diuretics therapy | 2.78 | 1.50 – 5.16 | 0.001 |  | |
| LGE= late gadolinium enhancement; LVEF=left ventricle ejection fraction; sPAP= systolic pulmonary artery pressure | | | | |  |

Table 2.c (suppl. data). Adjusted hazard ratios for death or new heart failure of the final model using MR derived left ventricle ejection fraction.

|  | **Adjusted HR** | **95% Confidence Interval** | **P value** |  | |
| --- | --- | --- | --- | --- | --- |
| LVEF (≤ 35%) | 4.89 | 2.59 – 9.27 | <0.0001 |  | |
| LGE total burden (≥ 45% of LV mass) | 3.32 | 1.64 – 6.71 | <0.001 |  | |
| Loop diuretics therapy | 3.22 | 1.72 – 6.05 | <0.001 |  | |
| Pulmonary hypertension (sPAP ≥ 35 mmHg) | 2.21 | 1.17 – 4.16 | 0.014 |  | |
| LGE= late gadolinium enhancement; LVEF=left ventricle ejection fraction; sPAP= systolic pulmonary artery pressure | | | | |  |
